# Special Issue “Sports Science in Children”

**DOI:** 10.3390/children11020202

**Published:** 2024-02-05

**Authors:** Diogo Coutinho, Bruno Travassos, Sara Santos, Pedro Figueiredo, Adam Leigh Kelly

**Affiliations:** 1 Department of Physical Education and Sports Sciences, University of Maia (UMAIA), 4475-690 Maia, Portugal; 2 Research Center in Sports Sciences, Health Sciences and Human Development, CIDESD, 5000-801 Vila Real, Portugal; bfrt@ubi.pt (B.T.); sdlsantos@utad.pt (S.S.); 3 Department of Sports Sciences, University of Beira Interior, 6201-001 Covilhã, Portugal; 4 Portugal Football School, Portuguese Football Federation, 5001-801 Oeiras, Portugal; 5 Department of Sports Sciences, Exercise and Health, University of Trás-os-Montes and Alto Douro, 5000-801 Vila Real, Portugal; 6 Physical Education Department, College of Education, United Arab Emirates University, Al Ain P.O. Box 15551, United Arab Emirates; pfigueiredo@uaeu.ac.ae; 7 Research for Athlete and Youth Sport Development (RAYSD) Lab, Research Centre for Life and Sport Sciences (CLaSS), Birmingham City University, Birmingham B15 3TN, UK; adam.kelly@bcu.ac.uk

## 1. Introduction

In recent times, research and technological advancements have opened an unprecedented window of opportunity for sports science to play a pivotal role in the holistic well-being of children [1]. By harnessing the power of sports science, we can not only design, implement, and evaluate sports programs for young athletes but also address broader aspects of their health [2]; therefore, the contemporary role of sports scientists in nurturing young talent cannot be understated, as they promote the most effective and efficient methods to support long-term sport and personal development [3]. By integrating sports science principles into children’s health initiatives, we not only contribute to long-term sustained athletic performance but also foster comprehensive health outcomes [4,5,6]. With appropriate immediate, short-, and long-term interventions, we can enhance athletes’ adaptability to navigate the complex and ever-changing competitive landscape.

In the spirit of knowledge dissemination, a diverse range of sports science topics was encouraged as a part of this Special Issue. This included topics such as understanding the various constraints of the performer (e.g., growth and maturation) [7,8], task (e.g., boundary conditions during small-sided games) [9,10], and environment (e.g., Relative Age Effects) [11,12] that coaches should consider in crafting dynamic learning environments; exploring innovative tools and instruments to assess athletes’ potential, development, and performance; and the influence of selection, training, and competition environments. Additionally, we sought perspectives and reviews capable of synthesizing knowledge, with a specific emphasis on enhancing our understanding of children’s health. This approach was intended to contribute not only to the refinement of youth sports practices but also to the development of comprehensive guidelines that encompass the broader spectrum of children’s well-being. Based on these objectives and as a conclusion to this Special Issue, this Editorial provides a general review of the studies and topics included, along with recommendations for future research and practice. In an attempt to view this research through the lens of knowledge mobilization (see [13,14]), we present this Editorial by exploring: (a) knowledge creation (i.e., the refinement of studies into knowledge tools or products through synthesizing our new knowledge), and (b) the shift from knowledge to action (i.e., the application of our new knowledge and skills through informing future policy and systemic change).

## 2. Knowledge Creation

This Special Issue features 18 manuscripts, including 14 empirical studies, two reviews, and two conceptual manuscripts. More than 9000 participants were included in the studies, which covered a variety of contexts, including team sports (soccer, n = 4; basketball, n = 2; rugby, n = 1; and volleyball, n = 1), individual sports (judo, n = 1, and tennis, n = 1), multiple sports (basketball, soccer, water polo, volleyball, and handball), and physical education (n = 2). This Special Issue stands as a notable catalyst for advancements within the field, stimulating and enriching meaningful discussions pertaining to the intersection of sports science and youth.

A central focus of this Special Issue was the Relative Age Effect (RAE) in sports. One study by Brustio et al. (1) demonstrated that relatively older players in the rugby union tend to achieve a high-level performance than their later-born counterparts, particularly in the positions of backs and forwards. Interestingly, this effect was more pronounced in players from southern regions, underscoring the impact of sociocultural influences on players’ career trajectories. The RAE was also observed by García-Rubio et al. (2) in soccer, spanning from under-17 (U17) to U21 players representing the youth national teams of Spain. Similar patterns were found by Andrew et al. (3), who investigated the RAE in soccer players of both genders across 55 associations under the UEFA governing body. They revealed an over-representation of male players born in the first quartile (January to March) in both the U17 and U19 categories, although these effects did not persist at the senior level. In opposition, no RAE was identified among female players at any level, which highlights that this phenomenon should be considered based on gender, age, competition level, and sport type.

A conceptual paper by Sweeney, Taylor, and MacNamara (4) offered an analysis of the RAE, emphasizing the importance of considering relative age and biological maturation from a holistic and individual perspective. There was an argument for the need for strategies to mitigate the effects of relative age and maturation that prioritize individual considerations, such as grouping players based on specific performance levels (e.g., technical skills) through approaches such as bio-banding rather than employing macro and meso methods. Regarding players’ biological maturation, Rüeger et al. (5) reviewed ultrasound-imaging-based methods for assessing biological maturation. While the study highlighted promising results, particularly in wrist and knee measurements due to their non-ionizing nature, affordability, and rapid results, the authors noted the diversity of methods used across studies, making it challenging to establish a “gold standard”. The study emphasized the need for further research standardizing procedures, given the potential impact of maturation on selection and career progression opportunities in sport. Collectively, these studies show how the RAE and maturity remain important topics in the sports science literature in children and helped to further understand the two different constructs and its applications.

Another central area of research in sports science involves the examination of young athletes’ anthropometric and physical capabilities. Larkin et al. (6) conducted a study comparing children’s height, body mass, sprinting, and jumping ability among talented youth athletes (identified at around 13 years old; n = 136) with the general youth population (n = 250). Their findings revealed that both male and female high-level athletes exhibited superior sprinting and jumping abilities compared to their counterparts at lower skill levels. Coincidentally, Pavlinovic et al. (7) suggest that the capacity to jump and sprint has been linked to reactive agility in early pubescent boys and girls (around 11 to 12 years old), a trait identified as pivotal for sporting success. In order to foster the development of reactive agility, pre-pubescent and early pubescent children must be exposed to diverse activities that enhance their fundamental movement skills (7).

Athletes’ physical performance, encompassing attributes such as speed, jumping abilities, and reactive agility, has emerged as a critical indicator of talent and a key factor in achieving a high performance (6, 7). In order to develop such physical skills, Xiao et al. (8) investigated the effects of a 12-week functional training intervention on adolescent Chinese tennis players. The authors documented significant enhancements in athletes’ strength and power, evidenced by improvements in exercises such as push-ups, medicine ball throws, and standing long jumps, favoring the group that underwent functional training over a control group. Taken together, these studies emphasize the value of implementing fitness testing in youth sports settings to monitor individuals’ physical development, as well as the importance of employing strength and conditioning coaches within youth sports environments (as part of an interdisciplinary team) to ensure that young athletes are adequately measured, trained, and developed. Indeed, these approaches should be considered based on the biological age of young athletes as well as their chronological age.

A comprehensive understanding of players’ developmental trajectories often requires a retrospective analysis of their career. In this context, Coutinho et al. (9) conducted an in-depth retrospective analysis of 546 young male athletes (aged 13.87 ± 1.9 years) across a range of sports who belong to regional sports clubs (n = 42). The study revealed that soccer players tended to specialize early despite engaging in a broader spectrum of team sports. Conversely, water polo players demonstrated a higher weekly training volume than soccer and handball by the age of 10 years. Despite this, these findings provide valuable insights to identify the specificities of each sport and unravel the complex factors that shape athletes’ pathways toward expertise.

In light of these multifaceted determinants that impact youth development, researchers have designed, implemented, and evaluated interventions to outline ways to nurture athletes’ progression in sports. For example, Côté et al. (10) examined the impact of the 1616 Story-Based Youth Development program in ice hockey. The results from the pilot study were encouraging, indicating that strategies of knowledge mobilization from other role models allow for the introduction of principles to sustain positive youth development. Practitioners should consider similar knowledge mobilization initiatives to enhance sports participation, reduce dropout rates, and foster positive attitudes toward sports among young athletes. Another approach to nurture athletes’ development involves exposing them to heightened levels of training and competition, such as playing with and against chronologically older opponents and participating in a greater number of competitions, e.g., [15]. Simenko (11) suggested that “playing-up”, commonly adopted in youth judo, may contribute to producing young medalists; however, they cautioned that this tactic should be viewed through a short-term lens, as in the long-term, it has been shown to elevate dropout rates in some instances. These findings underscore the importance of multifaceted interventions and holistic approaches in sports science to help shape and support the developmental pathways of young athletes.

As part of this Special Issue, some studies delved into strategies to better understand players’ technical and tactical skills in team sports. In this regard, Davids et al. (12) developed a paper describing the intricate interplay between individual, task, and environmental constraints, offering valuable insights into shaping learning opportunities. The work deepens the theoretical understanding and provides practical examples that coaches can employ to guide players’ interactions during practice. Additionally, the interaction between player performance and task conditions was extensively explored by Birrento Aguiar et al. (13), who conducted a review of various boundary condition manipulations in youth basketball players’ technical and tactical performances. While the research showcased diverse outcomes based on different manipulations, the authors highlighted the need for more studies with youth players to draw robust conclusions. They also emphasized the importance of studies comparing different age groups to offer crucial insights for training design.

In line with that, Tannoubi et al. (14) explored the impact of a video-modeling strategy on enhancing basketball skills in both open and closed tasks for youth players. Their findings indicated marked improvements in analytical drills (such as basketball skill tests) and small-sided games (three vs. three) among the group exposed to video modeling. This underscores the importance of coaches employing such methods to augment players’ learning experiences. Examining the nature of training tasks, Coutinho et al. (15) demonstrated that employing tasks grounded in cooperation and opposition led to superior outcomes in decision making and execution for U14 players when compared to more prescriptive tasks or those lacking opposition. Consequently, designing relevant learning environments is dependent on the strategies adopted by coaches as well as the type or interactions promoted by the training tasks.

Now, regarding the variation in individual constraints in players’ performance, Coito et al. (16) investigated the impact of players’ age (U15, U17, and U19) on the capacity of passing distances in soccer according to pitch zones (e.g., Z1 to Z6, in which Z1 refers to the areas closest to the team’s own goal, while Z6 refers to zones closest to the opponents’ goal). The research revealed that players tend to employ medium-distance passes close to their opponents’ targets and shorter passes in the midfielder zone of the pitch. Notably, older players demonstrated a propensity for medium and longer passes to more distant teammates, reflecting their heightened game knowledge. These findings shed light on the nuanced decision-making processes influenced by age and pitch positioning, guiding coaches in optimizing players’ strategic choices on the field.

To enhance the design of training tasks and facilitate continuous improvement among athletes, it is crucial to implement regular performance assessments. A study conducted with U13 female volleyball players demonstrated the effectiveness of adjusting the training focus based on a flexible periodization, tailored to players’ assessments during four vs. four small-sided game (SSG) formats, creating a more encouraging learning environment (Loureiro et al., 17). In contrast, adhering to a rigid planning structure whereby adjustments were only made monthly failed to align with the learners’ developmental needs. These findings emphasize the necessity for coaches and technical staff to consistently measure and assess players’ performance across all dimensions, enabling the creation of appropriate training tasks. In this context, the emergence of new instruments supporting the assessment of players’ technical and tactical skills during game-based situations is paramount. González-Rodenas et al. (18) developed the Observational Framework to Evaluate Individual Offensive Behavior in Youth Soccer (INDISOC). This instrument focuses on individual ball possession by evaluating players’ performance based on their ability to receive the ball, process, and execute the final action. The INDISOC serves as a valuable tool for coaches to measure players’ performance, enabling them to fine-tune the training process and enhance the overall training experience. Taken together, the technical and tactical development of athletes should be carefully considered by both researchers and practitioners and considered part of multidimensional training and research methodologies, respectively.

## 3. From Knowledge to Action

In this Special Issue, significant attention was given to the RAE and biological age in children’s sports, shedding light on the significant impact of birthdate on selection, development, and performance outcomes (1, 3, 4). Multifaceted interventions will play a pivotal role in nurturing athletes’ development and progression and ultimately will be crucial in moderating such effects. Practical implementation involves creating, adopting, and measuring strategies that tailor training and development programs based on specific ability levels rather than chronological age (4). This nuanced approach aims to mitigate the challenges posed by relative age and maturation differences and create a fairer and more individualized development pathway for young athletes that reflects the culture, sports, and individual differences.

Anthropometric and physical capabilities also emerged as critical indicators of talent in youth athletes (6–8) To translate this knowledge into practice, youth sports programs should incorporate regular fitness testing to monitor individual physical development. Additionally, emphasizing diverse activities for pre-pubescent and early pubescent children, can enhance fundamental movement skills, laying the foundation for improved sprinting, jumping, and agility abilities. It is noteworthy that the study of (7) revealed that change-of-direction abilities were significant predictors in both boys and girls, whereas jumping skills proved to be a determining factor exclusively in girls. These disparities appear to be linked to gender-specific patterns of sports involvement, with boys more frequently participating in team sports. Indeed, such gender-specific differences were noted by many authors across other disciplines in this Special Issue (e.g., differences in RAEs and maturation); therefore, sport scientists and other youth sport practitioners should be cautious of “copy–pasting” the norms of many male domains and ensure they meet the needs of the female athlete, particularly as both research and professional practice continue to rapidly grow in women’s and girls’ sport [16].

Technical and tactical skills in team sports can be effectively enhanced through thoughtful training task design and proper feedback strategies. Coutinho et al. (15) highlight the significance of training tasks that emphasize dynamic and unpredictable situations. This emphasizes the need for coaches to carefully consider the conditions introduced during practice sessions, ensuring a well-rounded approach to skill development; mainly when considering team sports in which the information that guides players’ actions is continuously changing as a result of the interaction between players and the environment. Complementarily, the introduction of new assessment instruments, such as INDISOC, provides coaches with valuable tools to measure, assess, and prescribe relevant training interventions that enhance players’ technical and tactical skills during soccer-game-based situations. In fact, coaches and sports scientists may use video-modeling strategies to augment players’ learning experiences (14). For example, coaches may use videos from role-models and high-level players to act as priming strategies that contributes to inspire players’ performance. For instance, the continuous performance assessment is critical for creating a conducive learning environment for young athletes. Accordingly, coaches should adjust training focus based on regular assessments by adopting a flexible periodization aligning with the developmental needs of players (17).

## 4. Future Directions

Building on the contemporary knowledge in sports science related to youth athletes, this Special Issue significantly contributes to the understanding of the pathways for talent development and the effective design of learning environments that concomitantly improves children health and well-being. While the insights provided are valuable, there is a need to continue exploring the evolution of sports coinciding with the mounting pressure on athletes to excel. For example, the potential negative impact of RAEs on numerous young athletes requires further research to mitigate such effects and help widen the pool of potential talent [17,18]. The negative effects of RAEs are also observed in school contexts, where children manifest lower satisfaction with their own life and lower health [19]. Although the impact of RAEs may be more pronounced in sports environments [18], these contexts simultaneously provide fertile ground for the development of strategies to alleviate their negative effects on children and youth [20]. Moreover, they offer an opportunity to enhance young individuals’ capacity to perceive and understand individual differences effectively. For that purpose, further studies may explore more individualized and holistic approaches (4). Incorporating assessments of players’ biological maturation and action capacities might offer part of the solution; however, despite promising results with ultrasound-image-based methods, the absence of standardized guidelines prevents these techniques from becoming the gold standard procedure (5). It is imperative for researchers to persistently emphasize and advance studies on biological maturity, recognizing the complex specificities inherent to each sport and acknowledging the diverse cultural backgrounds of the individuals involved, as this nuanced understanding is crucial for tailoring training programs and interventions that resonate with the unique physiological and sociocultural aspects of athletes [21].

While physical prowess is a paramount across sports, team sports also heavily rely on players’ technical and tactical capabilities. Recent research has explored how different boundary conditions influence player performance (13, 15, 16). However, many of these studies have taken a short-term perspective and focused on specific sample groups (such as amateur players, U19 players, or national-level athletes), limiting the generalizability of their findings. Future research endeavors should adopt medium- and long-term approaches, incorporating participants from diverse skill levels and age groups. Moreover, these interventions should include instruments for continuously monitoring players’ development, enabling the formulation of adaptable short-term plans that prioritize fostering positive and conducive learning environments. This holistic and inclusive approach promises to yield more comprehensive and actionable insights, propelling the field of youth sports science into a more nuanced and impactful future.

To our surprise, no studies on artificial intelligence (AI) were included. Indeed, AI (e.g., machine learning approaches and Generative AI) are becoming increasingly common in applied sport science environments, e.g., [22], thus capturing some of these stories and evaluating good practice will be important moving forward. For instance, the emergence of chatbots has played a significant role in facilitating interactions between AI and individuals [23]. One notable interaction involves requesting personalized training interventions, where AI provides suggestions and assistance at a personal level [24], potentially contributing to improved health and wellbeing [25]. This presents a realm of new opportunities to explore how AI can contribute to reducing the high prevalence of sedentary behavior among children [26] and addressing issues such as relative energy deficiency in sports (REDS) [27]. Consequently, there is a call to action to inspire researchers and stakeholders to intensify their efforts in developing experimental studies. These studies can contribute to the establishment of normative guidelines that assist children, youth, coaches, and parents in utilizing AI to promote an active lifestyle and enhance health and well-being.

It was also surprising to note that no study focused on children’s health nor well-being. In recent years, we have witnessed an unprecedented increase in childhood obesity [28]. This surge is compounded by elevated levels of sedentary behavior [29], extensive screen time [30] and decreased social interaction [31]. Collectively, these factors have contributed to a rise in issues related to mental health [32]. Indeed, mental health has garnered significant attention from researchers, particularly following the onset of the COVID-19 pandemic [33,34,35]. While it is well established that sports can positively impact mental health outcomes [36], additional research is needed to comprehend how children of various age groups and genders may respond to distinct training interventions from both micro- and macroscale perspectives. Consequently, future studies must delve into the ways in which sports sciences can actively contribute to the well-being of children, with a specific focus on mental health. This exploration should involve sharing insights from clinical, epidemiological, and translational science that are particularly relevant to children’s health.

Lastly, during sports science research and practice related to youth, we feel it is pertinent to highlight the need to ensure that we put the child before the athlete in all circumstances. More specifically, we encourage both researchers and practitioners to recognize the growing research in this field, as well as (and more importantly) the rapid increase in the professionalization of talent development systems (e.g., soccer academies), should not compromise children’s rights [37]. For instance, talent pathways are inherently judicious and discriminate due to the often subjective and selective nature of these processes, and thus, athletes regularly face various setbacks (e.g., deselection) and mistreatment (e.g., biases in selection). Researchers can negate this by ensuring organizations possess a clear and coherent children’s rights policy prior to engaging in research activities, whilst practitioners must respect and promote children’s rights, health, and wellbeing during applied activities.

## 5. Summary

This Special Issue was designed with the dual purpose of advancing youth sports science research to provide theoretical and practical insights. These insights not only contribute to enhancing the understanding of youth sports but also play a crucial role in promoting the health, both physical and mental, of children. The aim is to assist stakeholders and organizational structures in adapting their practices to foster more effective and efficient environments, with a specific emphasis on the significant impact these practices can have on the overall health and well-being of youth. Beyond expanding the knowledge in specific domains, research efforts have also aimed to raise new questions, inspiring future advancements in this field. Moving forward, researchers and practitioners are encouraged to co-create research projects to facilitate greater knowledge mobilization across children’s sports science. We extend heartfelt gratitude to all the contributing authors and reviewers whose dedication and expertise have made this research topic possible. Their invaluable contributions have enriched our understanding of sports science in youth and paved the way for innovative approaches and progressive developments in this crucial area. Thank you for your unwavering commitment to advancing the world of sports science and nurturing the talents of young athletes.

## References

[1] Radovic A., Badawy S.M. (2020). Technology Use for Adolescent Health and Wellness. Pediatrics.

[2] Smedegaard S., Christiansen L.B., Lund-Cramer P., Bredahl T., Skovgaard T. (2016). Improving the well-being of children and youths: A randomized multicomponent, school-based, physical activity intervention. BMC Public Health.

[3] Côté J., Turnnidge J., Vierimaa M., Smith A., Green K. (2016). A Personal Assets Approach to Youth Sport. Handbook of Youth Sport.

[4] Moeijes J., van Busschbach J.T., Bosscher R.J., Twisk J.W.R. (2018). Sports participation and psychosocial health: A longitudinal observational study in children. BMC Public Health.

[5] Kokko S. (2014). Sports clubs as settings for health promotion: Fundamentals and an overview to research. Scand. J. Public Health.

[6] Pate R.R., Hillman C.H., Janz K.F., Katzmarzyk P.T., Powell K.E., Torres A., Whitt-Glover M.C. (2019). Physical Activity and Health in Children Younger than 6 Years: A Systematic Review. Med. Sci. Sports Exerc..

[7] Malina R.M., Cumming S.P., Rogol A.D., Coelho E.S.M.J., Figueiredo A.J., Konarski J.M., Kozieł S.M. (2019). Bio-Banding in Youth Sports: Background, Concept, and Application. Sports Med..

[8] Towlson C., MacMaster C., Gonçalves B., Sampaio J., Toner J., MacFarlane N., Barrett S., Hamilton A., Jack R., Hunter F. (2022). The effect of bio-banding on technical and tactical indicators of talent identification in academy soccer players. Sci. Med. Footb..

[9] Clemente F.M., Ramirez-Campillo R., Sarmento H., Praça G.M., Afonso J., Silva A.F., Rosemann T., Knechtle B. (2021). Effects of Small-Sided Game Interventions on the Technical Execution and Tactical Behaviors of Young and Youth Team Sports Players: A Systematic Review and Meta-Analysis. Front. Psychol..

[10] Silva A.F., Ramirez-Campillo R., Sarmento H., Afonso J., Clemente F.M. (2021). Effects of Training Programs on Decision-Making in Youth Team Sports Players: A Systematic Review and Meta-Analysis. Front. Psychol..

[11] Wattie N., Schorer J., Baker J. (2015). The relative age effect in sport: A developmental systems model. Sports Med..

[12] Bozděch M., Agricola A., Zháněl J. (2023). The Relative Age Effect at Different Age Periods in Soccer: A Meta-Analysis. Percept. Mot. Ski..

[13] Graham I.D., Logan J., Harrison M.B., Straus S.E., Tetroe J., Caswell W., Robinson N. (2006). Lost in knowledge translation: Time for a map?. J. Contin. Educ. Health Prof..

[14] Kelly A.L., Turnnidge J. (2023). From Knowledge to Action: Bridging the Gap Between Research and Practice in Youth Soccer. Talent Identification and Development in Youth Soccer.

[15] Kelly A., Wilson M.R., Jackson D.T., Goldman D.E., Turnnidge J., Côté J., Williams C.A. (2021). A multidisciplinary investigation into “playing-up” in academy football according to age phase. J. Sports Sci..

[16] Kelly A.L., Brown T., Reed R., Côté J., Turnnidge J. (2022). Relative Age Effects in Male Cricket: A Personal Assets Approach to Explain Immediate, Short-Term, and Long-Term Developmental Outcomes. Sports.

[17] Kelly A.L., Calvo A.L., dos Santos S.D., Jiménez Sáiz S.L. (2022). Special Issue “Talent Identification and Development in Youth Sports”. Sports.

[18] Jones B.D., Lawrence G.P., Hardy L. (2018). New evidence of relative age effects in “super-elite” sportsmen: A case for the survival and evolution of the fittest. J. Sports Sci..

[19] Fumarco L., Baert S., Sarracino F. (2020). Younger, dissatisfied, and unhealthy—Relative age in adolescence. Econ. Hum. Biol..

[20] Webdale K., Baker J., Schorer J., Wattie N. (2020). Solving sport’s ‘relative age’ problem: A systematic review of proposed solutions. Int. Rev. Sport Exerc. Psychol..

[21] Malina R.M., Rogol A.D., Cumming S.P., Coelho e Silva M.J., Figueiredo A.J. (2015). Biological maturation of youth athletes: Assessment and implications. Br. J. Sports Med..

[22] Kelly A.L., Williams C.A., Cook R., Sáiz S.L.J., Wilson M.R. (2022). A Multidisciplinary Investigation into the Talent Development Processes at an English Football Academy: A Machine Learning Approach. Sports.

[23] Maher C.A., Davis C.R., Curtis R.G., Short C.E., Murphy K.J. (2020). A Physical Activity and Diet Program Delivered by Artificially Intelligent Virtual Health Coach: Proof-of-Concept Study. JMIR Mhealth Uhealth.

[24] Zhang J., Oh Y.J., Lange P., Yu Z., Fukuoka Y. (2020). Artificial Intelligence Chatbot Behavior Change Model for Designing Artificial Intelligence Chatbots to Promote Physical Activity and a Healthy Diet: Viewpoint. J. Med. Internet Res..

[25] Oh Y.J., Zhang J., Fang M.-L., Fukuoka Y. (2021). A systematic review of artificial intelligence chatbots for promoting physical activity, healthy diet, and weight loss. Int. J. Behav. Nutr. Phys. Act..

[26] Rodriguez-Ayllon M., Cadenas-Sánchez C., Estévez-López F., Muñoz N.E., Mora-Gonzalez J., Migueles J.H., Molina-García P., Henriksson H., Mena-Molina A., Martínez-Vizcaíno V. (2019). Role of Physical Activity and Sedentary Behavior in the Mental Health of Preschoolers, Children and Adolescents: A Systematic Review and Meta-Analysis. Sports Med..

[27] Ackerman K.E., Stellingwerff T., Elliott-Sale K.J., Baltzell A., Cain M., Goucher K., Fleshman L., Mountjoy M.L. (2020). #REDS (Relative Energy Deficiency in Sport): Time for a revolution in sports culture and systems to improve athlete health and performance. Br. J. Sports Med..

[28] Lanigan J., Barber S., Singhal A. (2010). Prevention of obesity in preschool children. Proc. Nutr. Soc..

[29] Saunders T.J., Chaput J.P., Tremblay M.S. (2014). Sedentary behaviour as an emerging risk factor for cardiometabolic diseases in children and youth. Can. J. Diabetes.

[30] Robinson T.N., Banda J.A., Hale L., Lu A.S., Fleming-Milici F., Calvert S.L., Wartella E. (2017). Screen Media Exposure and Obesity in Children and Adolescents. Pediatrics.

[31] Kraut R., Patterson M., Lundmark V., Kiesler S., Mukopadhyay T., Scherlis W. (1998). Internet paradox. A social technology that reduces social involvement and psychological well-being?. Am. Psychol..

[32] Membride H. (2016). Mental health: Early intervention and prevention in children and young people. Br. J. Nurs..

[33] Westrupp E.M., Bennett C., Berkowitz T., Youssef G.J., Toumbourou J.W., Tucker R., Andrews F.J., Evans S., Teague S.J., Karantzas G.C. (2023). Child, parent, and family mental health and functioning in Australia during COVID-19: Comparison to pre-pandemic data. Eur. Child Adolesc. Psychiatry.

[34] Ravens-Sieberer U., Erhart M., Devine J., Gilbert M., Reiss F., Barkmann C., Siegel N.A., Simon A.M., Hurrelmann K., Schlack R. (2022). Child and Adolescent Mental Health During the COVID-19 Pandemic: Results of the Three-Wave Longitudinal COPSY Study. J. Adolesc. Health.

[35] Elharake J.A., Akbar F., Malik A.A., Gilliam W., Omer S.B. (2023). Mental Health Impact of COVID-19 among Children and College Students: A Systematic Review. Child Psychiatry Hum. Dev..

[36] Belcher B.R., Zink J., Azad A., Campbell C.E., Chakravartti S.P., Herting M.M. (2021). The Roles of Physical Activity, Exercise, and Fitness in Promoting Resilience During Adolescence: Effects on Mental Well-Being and Brain Development. Biol. Psychiatry Cogn. Neurosci. Neuroimaging.

[37] Jayanthi N.A., Post E.G., Laury T.C., Fabricant P.D. (2019). Health Consequences of Youth Sport Specialization. J. Athl. Train..

